# Morphological Stasis in Wing Traits Despite Species Diversification in African and Malagasy *Miniopterus* Bats

**DOI:** 10.1002/ece3.73322

**Published:** 2026-03-27

**Authors:** Stefania Briones, Roberta Mason‐Gamer, Steven M. Goodman, Terrence C. Demos, Paul W. Webala, Bruce D. Patterson

**Affiliations:** ^1^ Negaunee Integrative Research Center Field Museum of Natural History Chicago Illinois USA; ^2^ Department of Biological Sciences University of Illinois at Chicago Chicago Illinois USA; ^3^ Department of Forestry and Wildlife Management Maasai Mara University Narok Kenya

**Keywords:** adaptive radiation, continental Africa, Madagascar, Miniopteridae, morphospace, wing morphology

## Abstract

Islands often give rise to adaptive radiations, owing to the absence of mainland competitors and predators. The long‐fingered bats (*Miniopterus*spp.) provide an opportunity to examine this pattern, as the genus includes sister radiations on Madagascar and on the African mainland. We measured wing elements related to flight in these two *Miniopterus* sister clades: one with 12 species from Madagascar and the other with nine species from Kenya, representing a comparable area of continental Africa. Principal component analysis revealed that *Miniopterus* species cluster within a narrow region of morphospace, with PC1 representing a size gradient that explains 93.6% of the variance in seven wing measurements. A phylomorphospace analysis integrating a resolved species phylogeny demonstrated that closely related species often occupy similar regions of morphospace, particularly among the smaller Malagasy taxa. Euclidean distance matrices showed similar nearest, average, and farthest neighbor values between Kenya and Madagascar, indicating strong morphological resemblance. Multivariate dispersion analysis yielded an observed mean dispersion of 1.8137, which did not significantly differ from a randomized expectation (*p* = 0.08819), suggesting that species are not more regularly or unevenly distributed than expected by chance. These findings indicate limited shape divergence in wing morphology between these two *Miniopterus* radiations. This work highlights the complexity of detecting adaptive patterns and suggests the need to incorporate broader ecological and behavioral data when studying diversification in bats.

## Graphical abstract

This study evaluates whether diversification in Malagasy and African *Miniopterus* is associated with divergence in linear wing morphology. Principal component and morphospace analyses indicate that wing morphology is highly conserved across species, with most variation reflecting differences in size rather than shape. These findings suggest that diversification in *Miniopterus* has occurred without strong divergence in wing morphology, highlighting the importance of considering additional ecological and behavioral axes when evaluating adaptive radiation.

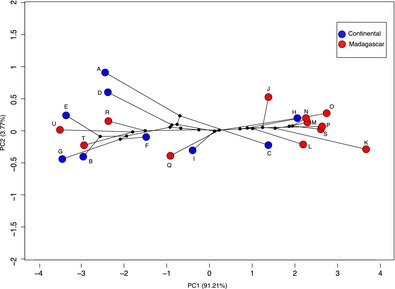

## 1 Introduction

Adaptive radiation is a fundamental evolutionary process that generates ecological and phenotypic diversity within rapidly diversifying lineages, often in response to new environmental opportunities (Schluter 2000). This process is based on common ancestry, phenotypic traits linked to ecological niches, functional advantages of the traits, and rapid speciation (Gavrilets and Losos 2009). While niche use is difficult to directly measure, morphological traits such as wing morphology have been shown to be reliable proxies for ecological niche and resource use in bats (Freeman 2000; Dumont et al. 2012). In bats, wing structure strongly influences flight performance, foraging behavior, and habitat use, making it a useful trait for studying evolutionary diversification (Norberg and Rayner 1987; Schoeman et al. 2015). Recent studies continue to support this approach, demonstrating that bat wing morphology can predict foraging strategy in certain insectivorous bat species (Fenton 1972; Jeyapraba et al. 2023; Veum et al. 2025).

Insectivorous bats are broadly categorized based on how they exploit aerial space while foraging. These categories include open‐space, clutter‐edge, and narrow‐space foragers (Schnitzler et al. 2003; Webala et al. 2019). Open‐space foragers have long, narrow wings suited for fast and sustained flight, while clutter‐edge and narrow‐space foragers have broader wings that allow for better maneuverability in complex environments. This further supports that wing morphology reflects the ecological niche used by bats employing different foraging strategies (Kalko et al. 2008; Norberg and Rayner 1987; Schoeman et al. 2015). Across sub‐Saharan Africa, diverse bat families occupy different foraging niches. However, Madagascar's bat fauna is less diverse in terms of clutter‐edge specialists, with families Nycteridae, Rhinolophidae, and Megadermatidae being absent from the island, while the clutter‐edge specialist family Miniopteridae (genus *Miniopterus* Bonaparte, 1837) is widespread and notably speciose on Madagascar and in sub‐Saharan Africa. These taxonomic differences provide the means to study whether reduced competition, reflected in the absence of other clutter‐edge specialist families, may have influenced morphological evolution in Malagasy *Miniopterus*, which have been shown to represent a monophyletic group (Demos et al. 2023).

*Miniopterus* is a genus of medium‐sized bats with a slender body and long, narrow, and bent wings; all members of the genus possess an elongated second phalanx of the third finger that also allows high maneuverability and greater aerodynamic efficiency (Norberg and Rayner 1987). This elongated second phalanx is unique among insectivorous bats, allowing them to forage both in open spaces and in clutter‐edge areas (Norberg and Rayner 1987; Denzinger and Schnitzler 2013; Vincent et al. 2011). There are currently 41 recognized species in the genus *Miniopterus* (Mammal Diversity Database 2026), all restricted to the Old World. Within the Afrotropical Realm, 10 species are found in sub‐Saharan Africa, 12 on Madagascar (two of which also inhabit the Comoro islands; Goodman et al. 2022), and one on São Tomé Island (Ibáñez and Juste 2019). A recent phylogeny of African *Miniopterus* based on ultra‐conserved elements (Demos et al. 2023) demonstrated monophyly of Madagascar's 12 *Miniopterus* species, indicating a single colonization of the island by this genus. Furthermore, it identified a well‐supported sister clade of 12 African *Miniopterus* that split from the Malagasy clade 9.06 Mya, diversifying in an environment with different ecological aspects, competitors, and resources (Demos et al. 2023). While the colonization and subsequent diversification of Madagascar's *Miniopterus* suggests an adaptive radiation (e.g., Christidis et al. 2014), it is unclear whether they exhibit the ecological and morphological diversification that is associated with this process. The same‐aged Malagasy and continental African sister clades of *Miniopterus* offer a means to evaluate this issue. Because the continental clade also radiated following their split, differences in wing morphology between these two regions might reflect differences in the ecological opportunities they subsequently encountered during the speciation process.

This study focuses on two central questions: (1) whether divergence in wing morphometrics in Malagasy *Miniopterus* is consistent with patterns expected under adaptive radiation following island colonization, and (2) whether disparity in linear wing traits differs between the Malagasy fauna and a mainland assemblage from a comparable area of sub‐Saharan Africa where additional clutter‐edge specialist bat families are present. Under this scenario, if adaptive divergence is expressed through linear wing traits, the Malagasy clade would be expected to exhibit greater morphological disparity in these traits than the African taxa, reflecting evolutionary responses to unoccupied ecological space and reduced interspecific competition.

Madagascar is known for its high levels of endemism and the distinct evolutionary pathways taken by many of its endemic species due to isolation and the availability of unoccupied ecological niches (Goodman 2023). Unlike the African mainland, where multiple bat families overlap and exploit clutter‐edge habitats, the extant bat fauna of Madagascar suggests that *Miniopterus* may have experienced reduced competition and expanded ecological opportunity. However, whether such opportunity translated into pronounced ecomorphological divergence remains unclear. To evaluate this, we quantified and compared wing morphology among Malagasy and sub‐Saharan Africa *Miniopterus* species. Continental representatives were collected in Kenya but belong to species that are widely distributed across sub‐Saharan Africa and are treated here as representative of the broader continental lineage. By explicitly comparing patterns of morphological dispersion and interspecific distances across regions, we assess whether divergence in linear wing morphology is consistent with adaptive diversification through wing traits or instead indicates a high degree of morphological conservatism.

## 2 Methods

### 2.1 Specimen Sampling and Morphological Measurements

*Miniopterus* specimens were selected from the mammal collection of the Field Museum of Natural History (Chicago), obtained during various expeditions between 1993 and 2019. We included only adult individuals, which presented fully fused epiphyseal joints, preserved originally in 10%–12% formaldehyde and then transferred to 70% ethanol; dry specimens were excluded due to the fragility of wing preparations and difficulty in measuring specific wing variables. The final dataset included 206 specimens spanning 21 taxonomic units: 12 species from the clade occurring on Madagascar (total surface area of 587,300 km^2^) and 9 species from Kenya (total surface area of 581,300 km^2^). Species included from each region and corresponding sample sizes are provided in Table 1. Although continental specimens were sampled from Kenya, these taxa are widely distributed across sub‐Saharan Africa and are treated here as representatives of the continental sister clade rather than of continental Africa as a whole. Among the mainland samples, four putative species have been included that are genetically distinct, substantiated by nuclear delimitation analyses and analyses of ultraconserved elements (Demos et al. 2023), but not yet formally described; these are referred to herein as *M*. clade 4, *M*. clade 5, *M*. clade 7, and *M*. clade 9 (Demos et al. 2020). It is important to note that species included in this study do not show signs of sexual dimorphism (McWilliam 1990; Goodman et al. 2009, 2011).

**TABLE 1:** *Miniopterus* species included in the study by locality with sample sizes.

Taxon	Region	*n*
*M. africanus*	Kenya	19
Clade 4	Kenya	5
Clade 5	Kenya	6
Clade 7	Kenya	6
Clade 9	Kenya	5
*M. fraterculus*	Kenya	10
*M. minor*	Kenya	20
*M. mossambicus*	Kenya	9
*M. natalensis*	Kenya	10
*M. aelleni*	Madagascar	9
*M. brachytragos*	Madagascar	8
*M. egeri*	Madagascar	11
*M. gleni*	Madagascar	10
*M. griffithsi*	Madagascar	9
*M. griveaudi*	Madagascar	20
*M. mahafaliensis*	Madagascar	6
*M. manavi*	Madagascar	13
*M. petersoni*	Madagascar	9
*M. sororculus*	Madagascar	10
*M. ambohitrensis*	Madagascar	6
*M. majori*	Madagascar	5

Seven morphological characters were measured for each specimen: tibia length (Tib), digit 2 metacarpal length (D2M), digit 3 metacarpal length (D3M), digit 3 phalanx 1 length (D3P1), digit 3 phalanx 2 length (D3P2), digit 4 metacarpal length (D4M), and digit 5 metacarpal length (D5M) (Belinne et al. 2024; Castillo‐Figueroa 2020) although these references used different measurement acronyms. All measurements were recorded to the nearest 0.01 mm using digital calipers and were taken by the same researcher to minimize recorder bias.

### 2.2 Statistical Analyses

All analyses were conducted using R (version 4.3.1, R Core Team 2024) and visualizations were produced using the *ggplot2* package (Wickham 2009). Prior to analysis, non‐numeric and descriptive columns were excluded, leaving a dataset of seven morphological variables. Standard data exploration was performed on the raw measurements to inspect data distributions, detect potential outliers, and ensure consistency across variables. These variables were scaled using the scale() function, and a principal component analysis (PCA) was conducted using the prcomp() function from base R (R Core Team 2024). PCA was performed to reduce the dimensionality of the morphological dataset and identify major axes of variation. PCA loadings and scores were extracted and stored in data frames for subsequent analysis. Species centroids were calculated as the mean position of individuals in morphospace, which provided a reference point for calculating morphological distances among species.

Euclidean distance matrices were calculated using the stats package, followed by a mean dispersion calculation to quantify the regularity of species spacing within morphospace (R Core Team 2024). Nearest and farthest neighbor distances were calculated to evaluate morphological clustering and potential ecological partitioning. Multivariate dispersion analysis (MVDISP) was performed to assess whether patterns of morphological variation among species reflect ecological pressures (Oksanen et al. 2020); specifically, this analysis evaluates whether species exhibit consistent spacing within morphospace, which may indicate adaptive responses or other ecological processes influencing their morphology (Shukla and Bhat 2017; Van de Perre et al. 2024).

To assess whether the observed dispersion pattern differed from random expectations, we generated 1000 randomized distance matrices by permuting the columns and rows of the original distance matrix. Mean dispersion was calculated for each randomized matrix to build a null distribution. The observed mean dispersion was then compared to this null distribution using a permutation test. A *p*‐value was calculated as the proportion of random dispersions that were greater or equal to the observed dispersion, implemented in R using the package *dplyr* (Wickham et al. 2026).

### 2.3 Phylomorphospace Analysis

To examine the relationship between morphology and evolutionary history, a phylomorphospace analysis was conducted using the phytools package (Revell 2024). As a first step, the phylogenetic tree generated by Demos et al. (2023) was pruned and reformatted using the consolidatespecies() function from the *ape* package in R (Paradis and Schliep 2019) to match the morphological dataset. Species that were not part of the monophyletic sister clades formed by the Malagasy and Kenyan lineages were removed from the analysis. Phylomorphospace integrates a phylogenetic tree with multivariate morphological data by projecting species centroids onto a shared morphospace. The visualization was plotted using the phylomorphospace() function with a “fan” configuration, allowing us to assess whether closely related species cluster together or occupy distinct regions of morphospace.

## 3 Results

### 3.1 Principal Component Analysis

**FIGURE 1:**
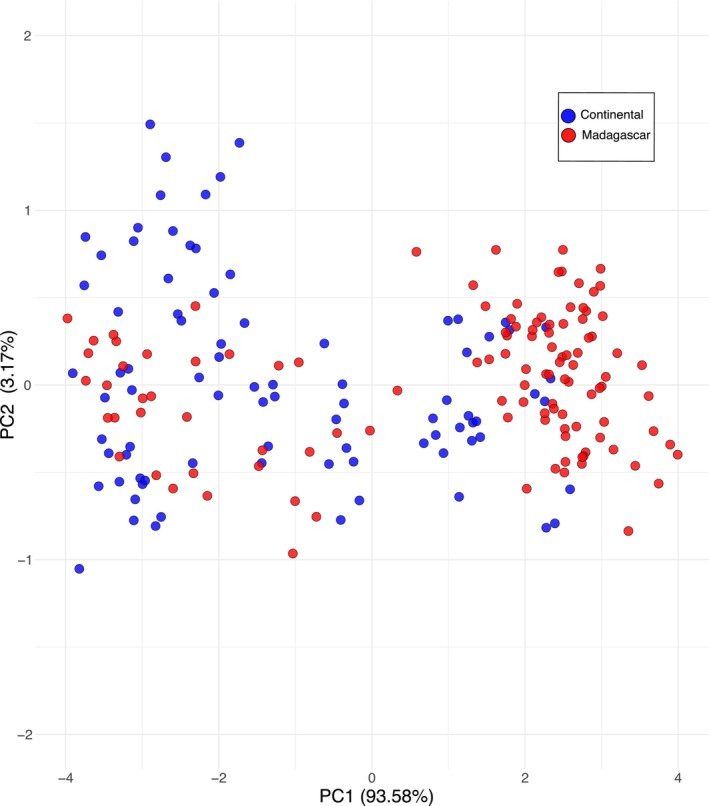
Principal component analysis (PCA) scatterplot of *Miniopterus* individuals from Kenya and Madagascar based on tibia and wing measurements. Kenya represents the continental African comparison. PC1 and PC2 explain 93.6% and 3.2% of the variance, respectively.

Principal component analysis (PCA) revealed clustering patterns among *Miniopterus* species based on morphological variation (Figure 1). Each species occupied a confined region of morphospace, and no taxon was widely dispersed. The first principal component (PC1) explained 93.6% of the variance in seven wing measurements and primarily reflected overall size variation among species, with larger species grouped towards the left side of the plot. The second principal component (PC2) accounted for an additional 3.2% of the variance.

**FIGURE 2:**
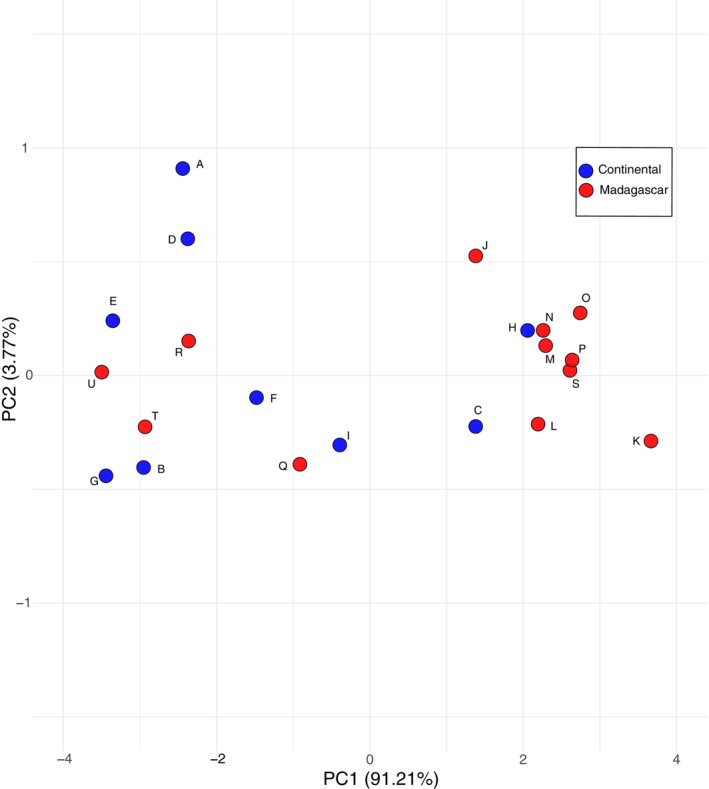
Principal Component Analysis (PCA) showing species centroid distribution in morphospace, categorized by locality. Malagasy species are compared with continental African species represented by samples from Kenya. Letters correspond to the following species: *M. fraterculus* (A), Clade 4 (B), *M. minor* (C), *M. natalensis* (D), Clade 5 (E), Clade 7 (F), *M. africanus* (G), Clade 9 (H), *M. mossambicus* (I), *M. ambohitrensis* (J), *M. brachytragos* (K), *M. aelleni* (L), *M. manavi* (M), *M. petersoni* (N), *M. mahafaliensis* (O), *M. griveaudi* (P), *M. sororculus* (Q), *M. majori* (R), *M. egeri* (S), *M. griffithsi* (T), and *M. gleni* (U). The unnamed clades are based on analyses presented in Demos et al. (2020).

In contrast, a PCA of species centroids explained 91.2% of the variance on PC1 and 3.8% on PC2, reflecting slightly reduced variation within species due to averaging. Species centroids occupied a similar region of morphospace and clustering patterns followed a consistent trend across continental and Malagasy species (Figure 2).

### 3.2 Phylomorphospace

**FIGURE 3:**
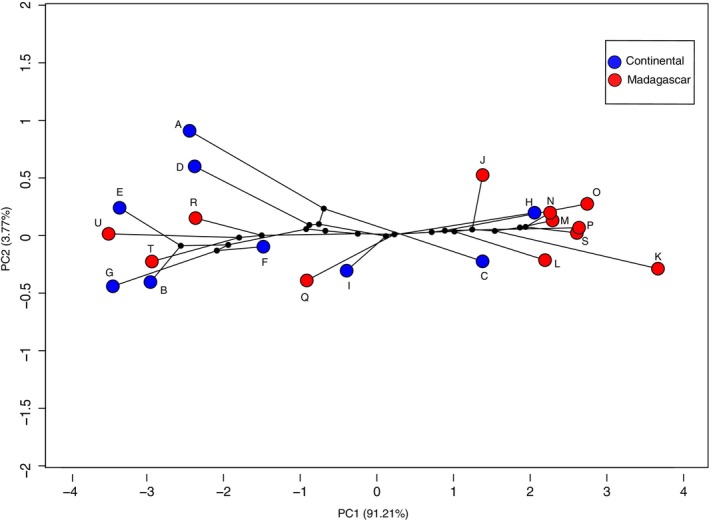
Phylomorphospace plot based on Principal Component Analysis (PCA) of wing morphology in *Miniopterus* species from Madagascar and continental Africa, represented here by Kenyan samples. Circles represent species centroids based on PCA scores. Black lines represent evolutionary relationships derived from the phylogenetic tree, and black dots indicate reconstructed ancestral nodes. Letters correspond to the following species: *M. fraterculus* (A), Clade 4 (B), *M. minor* (C), *M. natalensis* (D), Clade 5 (E), Clade 7 (F), *M. africanus* (G), Clade 9 (H), *M. mossambicus* (I), *M. ambohitrensis* (J), *M. brachytragos* (K), *M. aelleni* (L), *M. manavi* (M), *M. petersoni* (N), *M. mahafaliensis* (O), *M. griveaudi* (P), *M. sororculus* (Q), *M. majori* (R), *M. egeri* (S), *M. griffithsi* (T), and *M. gleni* (U). The unnamed clades are based on analyses presented in Demos et al. (2020).

The phylomorphospace analysis (Figure 3) integrated morphological data overlaid on phylogenetic relationships and examined if closely related species exhibited similar positions in morphological space. Certain closely related Malagasy species clustered together in morphospace, particularly among the smaller taxa. Other species occupied more distinctive positions, indicating variation in morphological space despite shared ancestry. No clear separation between continental and Malagasy species was observed.

### 3.3 Morphological Disparity and Dispersion

Euclidean distance matrices quantified pairwise morphological distances among species, providing a measure of interspecific differentiation within morphospace. Full distance matrices for each region are provided in Tables S1 and S2. Summary statistics for morphological distances in Continental and Malagasy assemblages are reported in Table S3. Nearest neighbor Euclidean distances were low for both continental and Malagasy species, with average values of 0.8075 and 0.5588 respectively. Mean pairwise distances were comparable between regions (continental: 2.6996, Madagascar: 2.7786), as were the farthest neighbor distances (continental: 5.6635, Madagascar: 6.3320). The observed mean dispersion was 1.8137, which did not differ significantly from a randomized expectation (*F* = 1.4918, *p* = 0.08819). This indicates that species level spacing in morphospace did not deviate from a random distribution.

## 4 Discussion

### 4.1 Morphological Differentiation and Dispersion

Our analyses document comparable patterns of variation in wing morphology among Malagasy and Kenyan *Miniopterus* species, with both groups occupying similar regions of morphospace. Despite occurring in an environment with fewer clutter‐edge specialist bat families, the Malagasy clade did not exhibit greater dispersion in wing morphospace than continental taxa, suggesting that the wing traits we examined did not differentially diverge in a competitively rarified environment. These results indicate a high degree of morphological conservatism in wing morphology. The measurements chosen all covary to such a degree that 93% of all variation is accounted for by the first principal component, often interpreted as size.

The conservative nature of wing morphology in *Miniopterus*, species essentially differing mainly in size rather than size and shape, is paralleled by comparable variation in their skull morphology. A recent analysis of 15 log‐transformed craniodental measurements of South Asian *Miniopterus* species found that 88.8% of overall variation was accounted by the first component (Kusuminda et al. 2022). As others have noted (e.g., Monadjem et al. 2020), morphological variation among *Miniopterus* species is largely limited to size variation. Genetic analyses are largely responsible for the nearly three‐fold increase in recognized *Miniopterus* species from 2005 (14 species; Simmons 2005) to the present (41; Simmons and Cirranello 2025).

Using a multivariate dispersion analysis of the Euclidean distance matrix, we found no significant deviation from random expectations of morphological dispersion. The observed mean dispersion (1.8137) did not differ from values generated under a null model (*p* = 0.08819), indicating overlap in wing morphospace among species. Because the measured wing traits primarily capture overall size variation, these results on their own cannot diagnose ecological niche differentiation or identify the evolutionary drivers underlying species diversification. Instead they demonstrate that diversification in *Miniopterus* has occurred without strong divergence in wing morphology. Similar outcomes have been documented in other Malagasy radiations, where geometric morphometric analyses revealed variation in evolutionary tempo and mode across traits, suggesting that adaptive patterning may depend on the traits and methods examined (Auerbach et al. 2025). These findings highlight that morphological conservatism in wing dimensions does not preclude diversification along other ecological or functional axes, which remain to be explored in this lineage.

### 4.2 Ecological Overlap and Niche Conservatism

The dominance of size variation does not contradict the observed conservatism in wing morphology but rather indicates that diversification in *Miniopterus* may occur primarily through size stratification within a broadly conserved wing structure. Adaptive radiations usually take place when species rapidly diversify after colonizing a new environment to exploit novel ecological niches (Schluter 2000). However, the morphological similarities among *Miniopterus* species suggest that the niches they occupy may not require significant or extreme morphological divergence. Although both Madagascar and Kenya encompass heterogeneous environments, many *Miniopterus* species seem to exploit similar clutter‐edge/edge‐space foraging niches within these diverse landscapes. This ecological overlap could diminish the selective pressures for morphological divergence with species retaining similar wing traits despite occupying different habitats (Fenton and Bogdanowicz 2002; Norberg and Rayner 1987; Schluter 2000).

Additionally, spatial factors such as distance to foraging resources and dispersal may result in relatively restricted species distributions which may strongly influence bat habitat use. This could potentially outweigh the selective pressure for morphological specialization (Rainho and Palmeirim 2011). Another contributing factor could be that clutter‐edge foraging habitats are broadly available across Madagascar, which may allow species, even in sympatry, to maintain a shared morphological outline. This phenomenon is consistent with the concept of niche conservatism, where species retain certain ancestral traits that remain well‐suited to specific environments, thus hindering the selective pressures for major morphological shifts despite the opportunities for diversification (Kelt et al. 2023; Losos 2010; Ranivo and Goodman 2007). Our results suggest that even across differing environments, wing traits among *Miniopterus* do not diverge strongly.

If *Miniopterus* species primarily forage in clutter‐edge habitats and are not strictly forest dependent (Kofoky et al. 2007; Randrianandrianina et al. 2006), the selective pressures to evolve distinct wing morphologies may be weak. While some degree of adaptation may occur to optimize foraging efficiency in these environments, the need for significant morphological differentiation could be minimal. The clustering of species within a narrow range in our morphospace (Figure 2) supports this interpretation, suggesting that evolutionary pressures to develop novel wing shapes are limited. This pattern implies that consistent environmental demands may be maintaining relatively uniform wing traits across species over time (Losos and Ricklefs 2009). This aligns with observations that across their Old World distribution, *Miniopterus* species exhibit consistent wing morphology, suggesting that these structures have remained evolutionarily conservative despite members of the genus occupying a broad geographic distribution and environments. However, when multiple *Miniopterus* species occur sympatrically, they are often stratified by size, with local faunas typically consisting of one or more small species, a medium‐sized species, and a large species (Monadjem et al. 2020). This pattern may be consistent with the evolutionary pressures to reduce interspecific competition for food resources through body size stratification, even as their wing shapes remain similar.

### 4.3 Wing Morphology as a Proxy for Ecology

We assumed the chosen wing measurements of *Miniopterus* species reflect broad aspects of their flight ecology despite lacking independent ecological data to test this relationship directly. This assumption is supported by comparative analyses performed on New World *Carollia perspicillata* (Phyllostomidae), which demonstrated a clear connection between wing morphology and flight performance (Carneiro et al. 2023). Wing length and width, determined by the relative proportions of the metacarpals and phalanges, influence the wing's shape and its ability to maneuver in cluttered environments (Castillo‐Figueroa 2018; Dietz et al. 2006; Findley et al. 1972). This relationship between morphological traits and habitat use has been a recurrent theme in Neotropical bats, where wing structure is strongly tied to foraging strategy and resource use (Castillo‐Figueroa 2020; Findley 1973, 1976). These dimensions contribute to wing loading and overall wing shape, which are critical factors in flight mechanics and habitat use (Castillo‐Figueroa 2020; Hedenström and Johansson 2015; Swartz and Middleton 2008).

The wing metrics analyzed here are expected to reflect broad, size‐related aspects of flight ecology, rather than direct measures of foraging performance or aerodynamic specialization. Because the linear wing measurements covary strongly, the first principal component primarily reflects overall size, with limited variation in shape. Our analyses assess whether diversification is expressed as divergence in size‐related wing dimensions that could alter broad flight constraints. Consequently, while these traits are ecologically informative, they cannot assess diversification along other ecological axes, including echolocation behavior or trophic specialization. Although a broad spectrum of studies involving bats suggests that wing elements should be ecologically informative and related to resource use, we are unaware of studies that directly evaluate the performance consequences of the traits we employed in *Miniopterus*. However, based on the linear measurements used here, we can state that the wing morphology of *Miniopterus* is highly conserved, varying principally in size, and that the Malagasy clade has diverged no more during their diversification than the Kenyan members of their sister clade on continental Africa.

### 4.4 Cryptic Ecological Divergence

Another possibility is that *Miniopterus* species exhibit cryptic ecological divergence not detectable through wing morphology. Differences in ear or tragus length and echolocation frequency could play a larger role in niche partitioning than wing shape or limb proportions (Calahorra‐Oliart et al. 2021; Losos 2010; Ramasindrazana et al. 2011). In addition, seasonal shifts in habitat and resource use may potentially reduce direct competition, as observed in other bat assemblages where intraspecific and interspecific interactions change over time (Aihartza et al. 2023; Raposeira et al. 2023). This behavioral plasticity could offer an alternative mechanism for niche partitioning that does not necessarily depend on specialized wing morphologies (Roeleke et al. 2018).

In addition, our analysis of morphospace included all members of the two sister clades occurring on Madagascar and in Kenya, respectively. It did not include partitioned analyses of the two‐to‐four species that locally coexist in each region and possibly compete. *Miniopterus inflatus* did not fall within the monophyletic sister clade of Malagasy *Miniopterus* and was therefore excluded from the analysis. It is important to mention that, of the 12 species of *Miniopterus* from Madagascar analyzed herein, nine have been described since 2007 (Goodman et al. 2010, 2011) and a few reputedly unnamed clades have been identified. In comparison, our Kenyan sample includes nine taxa, including several confirmed candidate species, from a region comparable in surface area to Madagascar (Demos et al. 2020, 2023). Coexisting sets of *Miniopterus* species are often size‐stratified (e.g., Monadjem et al. 2020), typically including one or more small species, a medium‐sized species, and a large species. This body size differentiation may help partition the resources they exploit and potentially reduce competition, despite similarities in their wing morphologies. Analysis of *Miniopterus* relationships at a finer spatial scale requires additional ecological information, such as roosting behavior, temporal activity patterns, and shifts in dietary composition (Tuneu‐Corral et al. 2025), but might illuminate the scale of interactions affecting *Miniopterus* evolution.

## 5 Conclusion

Based on the linear wing measurements used here, we find limited divergence in size‐based wing morphology among Malagasy *Miniopterus* and their continental sister clade. Interpreting these patterns in terms of ecological divergence expressed through linear wing traits remains tentative without additional ecological and functional data to test how these species interact with their environments. Data on trophic habits, activity periods, echolocation, and flight performance would strengthen inference about ecological differentiation and the processes underlying lineage diversification in future work. The Malagasy species provide a valuable counterpoint to well‐known island radiations that show clear adaptive diversification. They underscore the complexity of evolutionary responses to ecological opportunity and highlight that speciation and lineage diversification can proceed even in the absence of strong adaptive shifts in morphology.

## Author Contributions

**Stefania Briones:** data curation (lead), formal analysis (lead), investigation (equal), software (lead), visualization (lead), writing – original draft (lead), writing – review and editing (lead). **Roberta Mason‐Gamer:** formal analysis (supporting), investigation (equal), resources (equal), supervision (equal), writing – review and editing (equal). **Steven M. Goodman:** conceptualization (equal), data curation (equal), resources (equal), supervision (supporting), writing – review and editing (supporting). **Terrence C. Demos:** data curation (equal), resources (supporting), software (equal), writing – review and editing (supporting). **Paul W. Webala:** data curation (equal), resources (equal). **Bruce D. Patterson:** conceptualization (lead), data curation (equal), formal analysis (equal), investigation (equal), methodology (equal), resources (equal), supervision (lead), writing – review and editing (equal).

## Ethics Statement

The authors have nothing to report.

## Conflicts of Interest

The authors declare no conflicts of interest.

## Supporting information


**Table S1:** Euclidean distance matrix of species centroids in morphospace for Kenyan *Miniopterus* species.
**Table S2:** Euclidean distance matrix of species centroids in morphospace for Malagasy *Miniopterus* species.
**Table S3:** Summary of Euclidean distances among *Miniopterus* species from Kenya and Madagascar. Values represent mean pairwise distances, and smallest and largest observed interspecific distances with standard deviations.

## Data Availability

All morphological data, distance matrices, and R scripts used in this study are available on Zenodo: https://doi.org/10.5281/zenodo.17613323. The repository includes raw measurement data, PCA and dispersion analysis code, and supporting files used to generate figures and statistical results.

## References

[ece373322-bib-0001] Aihartza, J. , N. Vallejo , M. Aldasoro , et al. 2023. “Aerospace‐Foraging Bats Eat Seasonably Across Varying Habitats.” Scientific Reports 13, no. 1: 19576. 10.1038/s41598-023-46939-7. 37950015 PMC10638376

[ece373322-bib-0002] Auerbach, A. L. B. , E. H. J. Lim , and S. Reddy . 2025. “Tempo and Mode of Evolution Across Multiple Traits in an Adaptive Radiation of Birds (Vangidae).” Evolution 79, no. 9: 1710–1726. 10.1093/evolut/qpaf117. 40434827

[ece373322-bib-0003] Belinne, H. G. , S. C. Vrla , J. E. Czap , and R. D. Stevens . 2024. “Wing Measurements for Differentiating Three Cryptic Species of *Myotis* (Mammalia: Chiroptera) That Co‐Occur in the Southeastern United States.” Occasional Papers, the Museum, Texas Tech University 388, no. 1: 1–11.

[ece373322-bib-0063] Calahorra‐Oliart, A. , S. M. Ospina‐Garcés , and L. León‐Paniagua . 2021. “Cryptic Species in *Glossophaga soricina* (Chiroptera: Phyllostomidae): Do Morphological Data Support Molecular Evidence?” Journal of Mammalogy 102, no. 1: 54–68.

[ece373322-bib-0004] Carneiro, L. O. , B. Mellado , M. R. Nogueira , A. P. Cruz‐Neto , and L. R. Monteiro . 2023. “Flight Performance and Wing Morphology in the Bat *Carollia perspicillata*: Biophysical Models and Energetics.” Integrative Zoology 18, no. 6: 876–890. 10.1111/1749-4877.12707. 36610047

[ece373322-bib-0005] Castillo‐Figueroa, D. 2018. “Fluctuating Asymmetry of Three Bat Species in Extensive Livestock Systems of Córdoba Department, Colombia.” Revista Colombiana de Ciencia Animal 10, no. 2: 143–153. 10.24188/recia.v10.n2.2018.623.

[ece373322-bib-0006] Castillo‐Figueroa, D. 2020. “Ecological Morphology of Neotropical Bat Wing Structures.” Zoological Studies 59: e60. 10.6620/ZS.2020.59-60. 34140977 PMC8181164

[ece373322-bib-0007] Christidis, L. , S. M. Goodman , K. Naughton , and B. Appleton . 2014. “Insights Into the Evolution of a Cryptic Radiation of Bats: Dispersal and Ecological Radiation of Malagasy *Miniopterus* (Chiroptera: Miniopteridae).” PLoS One 9, no. 3: e92440. 10.1371/journal.pone.0092440. 24642892 PMC3958536

[ece373322-bib-0008] Demos, T. C. , P. W. Webala , S. M. Goodman , et al. 2023. “Ultraconserved Elements Resolve Phylogenetic Relationships and Biogeographic History of African–Malagasy Bent‐Winged Bats (*Miniopterus*).” Molecular Phylogenetics and Evolution 188: 107890. 10.1016/j.ympev.2023.107890. 37517508

[ece373322-bib-0009] Demos, T. C. , P. W. Webala , H. L. Lutz , et al. 2020. “Multilocus Phylogeny of a Cryptic Radiation of Afrotropical Long‐Fingered Bats (Chiroptera: Miniopteridae).” Zoologica Scripta 49, no. 1: 1–13. 10.1111/zsc.12388.

[ece373322-bib-0064] Denzinger, A. , and H. Schnitzler . 2013. “Bat Guilds, a Concept to Classify the Highly Diverse Foraging and Echolocation Behaviors of Microchiropteran Bats.” Frontiers in Physiology 4: 164. 10.3389/fphys.2013.00164. 23840190 PMC3699716

[ece373322-bib-0010] Dietz, C. , I. Dietz , and B. M. Siemers . 2006. “Wing Measurement Variations in the Five European Horseshoe Bat Species (Chiroptera: Rhinolophidae).” Journal of Mammalogy 87, no. 6: 1241–1251. 10.1644/05-MAMM-A-299R2.1.

[ece373322-bib-0011] Dumont, E. R. , L. M. Dávalos , A. Goldberg , S. E. Santana , K. Rex , and C. C. Voigt . 2012. “Morphological Innovation, Diversification, and Invasion of a New Adaptive Zone.” Proceedings of the Royal Society B: Biological Sciences 279, no. 1734: 1797–1805. 10.1098/rspb.2011.2005. PMC3297451 22113035

[ece373322-bib-0012] Fenton, M. B. 1972. “The Structure of Aerial‐Feeding Bat Faunas as Indicated by Ears and Wing Elements.” Canadian Journal of Zoology 50, no. 3: 287–296. 10.1139/z72-039.

[ece373322-bib-0014] Fenton, M. B. , and W. Bogdanowicz . 2002. “Relationships Between External Morphology and Foraging Behaviour: Bats in the Genus *Myotis* .” Canadian Journal of Zoology 80, no. 6: 1004–1013. 10.1139/z02-083.

[ece373322-bib-0015] Findley, J. S. 1973. “Phenetic Packing as a Measure of Faunal Diversity.” American Naturalist 107, no. 956: 580–584. 10.1086/282860.

[ece373322-bib-0016] Findley, J. S. 1976. “The Structure of Bat Communities.” American Naturalist 110, no. 971: 129–139.

[ece373322-bib-0017] Findley, J. S. , E. H. Studier , and D. E. Wilson . 1972. “Morphological Properties of Bat Wings.” Journal of Mammalogy 53, no. 3: 429–444. 10.2307/1379035.

[ece373322-bib-0018] Freeman, P. 2000. “Macroevolution in Microchiroptera: Recoupling Morphology and Ecology With Phylogeny.” Evolutionary Ecology Research 2, no. 3: 317–335.

[ece373322-bib-0019] Gavrilets, S. , and J. B. Losos . 2009. “Adaptive Radiation: Contrasting Theory With Data.” Science 323, no. 5915: 732–737. 10.1126/science.1157966. 19197052

[ece373322-bib-0020] Goodman, S. M. 2023. “Updated Estimates of Biotic Diversity and Endemism for Madagascar—Revisited After 20 Years.” Oryx 57, no. 5: 561–565. 10.1017/S0030605322001284.

[ece373322-bib-0021] Goodman, S. M. , C. P. Maminirina , H. M. Bradman , L. Christidis , and B. R. Appleton . 2009. “The Use of Molecular Phylogenetic and Morphological Tools to Identify Cryptic and Paraphyletic Species: Examples From the Diminutive Long‐Fingered Bats (Chiroptera: Miniopteridae: *Miniopterus*) on Madagascar.” American Museum Novitates 3669: 1–34. 10.1206/652.1.

[ece373322-bib-0022] Goodman, S. M. , C. P. Maminirina , H. M. Bradman , L. Christidis , and B. R. Appleton . 2010. “Patterns of Morphological and Genetic Variation in the Endemic Malagasy Bat *Miniopterus gleni* (Chiroptera: Miniopteridae), With the Description of a New Species, *M. griffithsi* .” Journal of Zoological Systematics and Evolutionary Research 48: 75–86. 10.1111/j.1439-0469.2009.00524.x.

[ece373322-bib-0023] Goodman, S. M. , B. Ramasindrazana , C. P. Maminirina , M. C. Schoeman , and B. Appleton . 2011. “Morphological, Bioacoustical, and Genetic Variation in *Miniopterus* Bats From Eastern Madagascar, With the Description of a New Species.” Zootaxa 2880, no. 1: 1–19. 10.11646/zootaxa.2880.1.1.

[ece373322-bib-0024] Goodman, S. M. , B. Ramasindrazana , and M. C. Schoeman . 2022. “Bats of Madagascar.” In The New Natural History of Madagascar, edited by S. M. Goodman , 1894–1911. Princeton University Press.

[ece373322-bib-0025] Hedenström, A. , and L. C. Johansson . 2015. “Bat Flight: Aerodynamics, Kinematics and Flight Morphology.” Journal of Experimental Biology 218, no. 5: 653–663. 10.1242/jeb.031203. 25740899

[ece373322-bib-0026] Ibáñez, C. , and J. Juste . 2019. “Family Miniopteridae (Long‐Fingered Bats).” In Handbook of the Mammals of the World, edited by D. E. Wilson and R. A. Mittermeier , vol. 9 Bats, 674–709. Lynx Ediciones.

[ece373322-bib-0027] Jeyapraba, L. , I. V. Margaret , D. Addline , and V. Sakthi . 2023. “Prediction of Foraging Strategy of Insectivorous Bats Through Their Wing Morphology.” Journal of Survey in Fisheries Sciences 10, no. 3S: 1903–1917. 10.17762/sfs.v10i3S.695.

[ece373322-bib-0028] Kalko, E. K. V. , S. Estrada Villegas , M. Schmidt , M. Wegmann , and C. F. J. Meyer . 2008. “Flying High—Assessing the Use of the Aerosphere by Bats.” Integrative and Comparative Biology 48, no. 1: 60–73. 10.1093/icb/icn030. 21669773

[ece373322-bib-0029] Kelt, D. A. , S. A. Coppeto , D. H. Van Vuren , J. Sullivan , J. A. Wilson , and N. Reid . 2023. “Niche Conservatism Versus Niche Differentiation in Sympatric Chipmunks in the Northern Sierra Nevada.” Journal of Mammalogy 104, no. 4: 979–992. 10.1093/jmammal/gyad048.

[ece373322-bib-0030] Kofoky, A. , D. Andriafidison , F. Ratrimomanarivo , et al. 2007. “Habitat Use, Roost Selection and Conservation of Bats in Tsingy de Bemaraha National Park, Madagascar.” Biodiversity and Conservation 16, no. 4: 1039–1053. 10.1007/s10531-006-9071-0.

[ece373322-bib-0031] Kusuminda, T. , A. Mannakkara , K. D. B. Ukuwela , et al. 2022. “DNA Barcoding and Morphological Analyses Reveal a Cryptic Species of *Miniopterus* From India and Sri Lanka.” Acta Chiropterologica 24, no. 1: 1–17. 10.3161/15081109ACC2022.24.1.001.

[ece373322-bib-0032] Losos, J. B. 2010. “Adaptive Radiation, Ecological Opportunity, and Evolutionary Determinism. American Society of Naturalists E. O. Wilson Award Address.” American Naturalist 175: 623–639. 10.1086/652433. 20412015

[ece373322-bib-0033] Losos, J. B. , and R. E. Ricklefs . 2009. “Adaptation and Diversification on Islands.” Nature 457, no. 7231: 830–836. 10.1038/nature07893. 19212401

[ece373322-bib-0034] Mammal Diversity Database . 2026. “Mammal Diversity Database (Version 2.0).” Zenodo. 10.5281/zenodo.4139818.

[ece373322-bib-0035] McWilliam, A. N. 1990. “Mating System of the Bat *Miniopterus minor* (Chiroptera: Vespertilionidae) in Kenya, East Africa: A Lek?” Ethology 85: 302–312. 10.1111/j.1439-0310.1990.tb00409.

[ece373322-bib-0036] Monadjem, A. , J. T. Shapiro , L. R. Richards , et al. 2020. “Systematics of West African *Miniopterus* With the Description of a New Species.” Acta Chiropterologica 21, no. 2: 237–256. 10.3161/15081109ACC2019.21.2.001.

[ece373322-bib-0037] Norberg, U. M. , and J. M. V. Rayner . 1987. “Ecological Morphology and Flight Adaptations in Bats (Mammalia; Chiroptera): Wing Adaptations, Flight Performance, Foraging Strategy and Echolocation.” Philosophical Transactions of the Royal Society of London. B, Biological Sciences 316: 335–427. 10.1098/rstb.1987.0030.

[ece373322-bib-0038] Oksanen, J. , F. G. Blanchet , M. Friendly , et al. 2020. “Vegan: Community Ecology Package (Version 2.5‐7) [R Package].” https://CRAN.R‐project.org/package=vegan.

[ece373322-bib-0039] Paradis, E. , and K. Schliep . 2019. “Ape 5.0: An Environment for Modern Phylogenetics and Evolutionary Analyses in R.” Bioinformatics 35, no. 3: 526–528. 10.1093/bioinformatics/bty633. 30016406

[ece373322-bib-0040] R Core Team . 2024. R: A Language and Environment for Statistical Computing. R Foundation for Statistical Computing. https://www.r‐project.org/.

[ece373322-bib-0041] Rainho, A. , and J. M. Palmeirim . 2011. “The Importance of Distance to Resources in the Spatial Modelling of Bat Foraging Habitat.” PLoS One 6, no. 4: e19227. 10.1371/journal.pone.0019227. 21547076 PMC3081845

[ece373322-bib-0042] Ramasindrazana, B. , S. M. Goodman , M. C. Schoeman , and B. Appleton . 2011. “Identification of Cryptic Species of *Miniopterus* Bats (Chiroptera: Miniopteridae) From Madagascar and the Comoros Using Bioacoustics Overlaid on Molecular Genetic and Morphological Characters.” Biological Journal of the Linnean Society 104, no. 2: 284–302. 10.1111/j.1095-8312.2011.01740.x.

[ece373322-bib-0043] Randrianandrianina, F. , D. Andriafidison , A. Kofoky , et al. 2006. “Habitat Use and Conservation of Bats in Rainforest and Adjacent Human‐Modified Habitats in Eastern Madagascar.” Biodiversity and Conservation 15, no. 9: 2817–2838. 10.1007/s10531-005-5407-5.

[ece373322-bib-0044] Ranivo, J. , and S. M. Goodman . 2007. “Patterns of Ecomorphological Variation in the Bats of Western Madagascar: Comparisons Among and Between Communities Along a Latitudinal Gradient.” Mammalian Biology 72, no. 1: 1–13. 10.1016/j.mambio.2006.08.004.

[ece373322-bib-0045] Raposeira, H. , P. Horta , R. Heleno , and H. Rebelo . 2023. “Changing With the Times: Seasonal Environmental Gradients Unveil Dynamic Bat Assemblages and Vulnerability.” Ecology and Evolution 13: e10246. 10.1002/ece3.10246. 37470030 PMC10352094

[ece373322-bib-0046] Revell, L. J. 2024. “Phytools 2.0: An Updated R Ecosystem for Phylogenetic Comparative Methods (and Other Things).” PeerJ 12: e16505. 10.7717/peerj.16505. 38192598 PMC10773453

[ece373322-bib-0047] Roeleke, M. , L. Johannsen , and C. C. Voigt . 2018. “How Bats Escape the Competitive Exclusion Principle—Seasonal Shift From Intraspecific to Interspecific Competition Drives Space Use in a Bat Ensemble.” Frontiers in Ecology and Evolution 6: 101. 10.3389/fevo.2018.00101.

[ece373322-bib-0048] Schluter, D. 2000. The Ecology of Adaptive Radiation. Oxford University Press.

[ece373322-bib-0049] Schnitzler, H. U. , C. F. Moss , and A. Denzinger . 2003. “From Spatial Orientation to Food Acquisition in Echolocating Bats.” Trends in Ecology & Evolution 18, no. 8: 386–394. 10.1016/S0169-5347(03)00185-X.

[ece373322-bib-0050] Schoeman, M. C. , S. M. Goodman , B. Ramasindrazana , D. Koubínová , and B. Appleton . 2015. “Species Interactions During Diversification and Community Assembly in Malagasy *Miniopterus* Bats.” Evolutionary Ecology 29: 17–47. 10.1007/s10682-014-9745-4.

[ece373322-bib-0051] Shukla, R. , and A. Bhat . 2017. “Morphological Divergences and Ecological Correlates Among Wild Populations of Zebrafish ( *Danio rerio* ).” Environmental Biology of Fishes 100: 251–264. 10.1007/s10641-017-0576-3.

[ece373322-bib-0052] Simmons, N. B. 2005. “Chiroptera.” In Mammal Species of the World: A Taxonomic and Geographic Reference, edited by D. E. Wilson and D. A. M. Reeder , vol. 1, 3rd ed., 312–529. Johns Hopkins University Press.

[ece373322-bib-0053] Simmons, N. B. , and A. L. Cirranello . 2025. “Bat Species of the World: A Taxonomic and Geographic Database (Version 1.9) [Database].” https://batnames.org/.

[ece373322-bib-0054] Swartz, S. M. , and K. M. Middleton . 2008. “Biomechanics of the Bat Limb Skeleton: Scaling, Material Properties and Mechanics.” Cells, Tissues, Organs 187: 59–84. 10.1159/000109964. 18160803

[ece373322-bib-0055] Tuneu‐Corral, A. , C. Andrianalijaona , F. D. Benirina , et al. 2025. “Beyond Borders: The Role of Protected Areas in Promoting Bat‐Mediated Pest Suppression in Rural Areas of Madagascar.” Agriculture, Ecosystems & Environment 387: 109590. 10.1016/j.agee.2025.109590.

[ece373322-bib-0056] Van de Perre, F. , M. R. Willig , S. J. Presley , H. Leirs , and E. Verheyen . 2024. “The Structure of Congolese Shrew Ensembles: Competition and Spatial Variation in Resource Abundance.” Journal of Mammalogy 105, no. 5: 1083–1093. 10.1093/jmammal/gyae032.

[ece373322-bib-0057] Veum, S. A. , A. K. Tallon , and S. A. Rush . 2025. “Trophic Niche Partitioning Among Insectivorous Bats Using a Combination of Stable Isotope Analyses and Wing Morphology.” Acta Chiropterologica 27, no. 1: 39–52. 10.3161/15081109acc2025.27.1.004.

[ece373322-bib-0058] Vincent, S. , M. Nemoz , and S. Aulagnier . 2011. “Activity and Foraging Habitats of *Miniopterus schreibersii* (Chiroptera: Miniopteridae) in Southern France: Implications for Its Conservation.” Hystrix 22, no. 1: 69–77. 10.4404/hystrix-22.1-4524.

[ece373322-bib-0059] Webala, P. W. , J. Mwaura , J. M. Mware , G. G. Ndiritu , and B. D. Patterson . 2019. “The Effect of Habitat Fragmentation on the Bats of Kakamega Forest, Western Kenya.” Journal of Tropical Ecology 35, no. 6: 260–269. 10.1017/S0266467419000221.

[ece373322-bib-0060] Wickham, H. 2009. ggplot2: Elegant Graphics for Data Analysis. Springer.

[ece373322-bib-0062] Wickham, H. , R. François , L. Henry , K. Müller , and D. Vaughan . 2026. “dplyr: A Grammar of Data Manipulation.” R Package Version 1.2.0. https://dplyr.tidyverse.org.

