# Supplementary material for: Morphological Stasis in Wing Traits Despite Species Diversification in African and Malagasy Miniopterus Bats
Source: Ecol Evol. 2026 Mar 27;16(4):e73322. doi: 10.1002/ece3.73322 (PMC13107277; doi:10.1002/ece3.73322)
Supplement: Supplementary file 1 — Table S1: Euclidean distance matrix of species centroids in morphospace for Kenyan Miniopterus species. Table S2: Euclidean distance matrix of species centroids in morphospace for Malagasy Miniopterus species. Table S3: Summary of Euclidean distances among Miniopterus species from Kenya and Madagascar. Values represent mean pairwise distances, and smallest and largest observed interspecific distances with standard deviations. [file ECE3-16-e73322-s001.docx]

**Supporting Information**

**Table** **S1.** Euclidean distance matrix of species centroids in morphospace for Kenyan Miniopterus species

| **Taxon** | ***M. africanus*** | **Clade 4** | **Clade 5** | **Clade 7** | **Clade 9** | ***M. fraterculus*** | ***M. minor*** | ***M. mossambicus*** | ***M. natalensis*** |
| --- | --- | --- | --- | --- | --- | --- | --- | --- | --- |
| ***M. africanus*** | 0 | 0.7248 | 1.2986 | 2.5218 | 6.7196 | 1.9722 | 5.8693 | 3.7852 | 1.7941 |
| **Clade 4** | 0.7248 | 0 | 1.2298 | 1.8016 | 5.9971 | 1.5142 | 5.1445 | 3.0606 | 1.2431 |
| **Clade 5** | 1.2986 | 1.2298 | 0 | 2.2457 | 6.2569 | 0.9883 | 5.5056 | 3.5482 | 1.0635 |
| **Clade 7** | 2.5218 | 1.8016 | 2.2457 | 0 | 4.1986 | 1.5043 | 3.3630 | 1.3170 | 1.2256 |
| **Clade 9** | 6.7196 | 5.9971 | 6.2569 | 4.1985 | 0 | 5.2993 | 0.9414 | 2.9778 | 5.2032 |
| ***M. fraterculus*** | 1.9722 | 1.5141 | 0.9882 | 1.5043 | 5.2993 | 0 | 4.5802 | 2.7178 | 0.3671 |
| ***M. minor*** | 5.8693 | 5.1444 | 5.5055 | 3.3630 | 0.9414 | 4.5802 | 0 | 2.0910 | 4.4423 |
| ***M. mossambicus*** | 3.7852 | 3.0606 | 3.5481 | 1.3169 | 2.9778 | 2.7178 | 2.0910 | 0 | 2.4995 |
| ***M. natalensis*** | 1.7941 | 1.2431 | 1.0634 | 1.2255 | 5.2032 | 0.3671 | 4.4425 | 2.4995 | 0 |

| **Taxon** | ***M. aelleni*** | ***M. ambohitrensis*** | ***M. brachytragos*** | ***M. egeri*** | ***M. gleni*** | ***M. griffithsi*** | ***M. griveaudi*** | ***M. mahafaliensis*** | ***M. majori*** | ***M. manavi*** | ***M. petersoni*** | ***M. sororculus*** |
| --- | --- | --- | --- | --- | --- | --- | --- | --- | --- | --- | --- | --- |
| ***M. aelleni*** | 0 | 0.9424 | 1.5634 | 0.4579 | 5.9979 | 5.4215 | 0.5365 | 0.951 | 4.8114 | 0.1405 | 0.2304 | 3.2864 |
| ***M. ambohitrensis*** | 0.9424 | 0 | 2.4958 | 1.3958 | 5.1174 | 4.5421 | 1.4719 | 1.6811 | 3.947 | 1.0071 | 1.1382 | 2.4387 |
| ***M. brachytragos*** | 1.5634 | 2.4958 | 0 | 1.1055 | 7.5471 | 6.9711 | 1.0271 | 1.4518 | 6.3548 | 1.4888 | 1.3576 | 4.8285 |
| ***M. egeri*** | 0.4579 | 1.3958 | 1.1055 | 0 | 6.4501 | 5.8737 | 0.08 | 0.8603 | 5.2612 | 0.398 | 0.2689 | 3.7351 |
| ***M. gleni*** | 5.9979 | 5.1174 | 7.5471 | 6.4501 | 0 | 0.5764 | 6.53 | 6.3265 | 1.1999 | 6.0944 | 6.2196 | 2.7203 |
| ***M. griffithsi*** | 5.4215 | 4.5421 | 6.9711 | 5.8737 | 0.5764 | 0 | 5.9536 | 5.757 | 0.6338 | 5.5181 | 5.6432 | 2.1454 |
| ***M. griveaudi*** | 0.5365 | 1.4719 | 1.0271 | 0.08 | 6.53 | 5.9536 | 0 | 0.8847 | 5.3412 | 0.4696 | 0.3385 | 3.8152 |
| ***M. mahafaliensis*** | 0.951 | 1.6811 | 1.4518 | 0.8603 | 6.3265 | 5.757 | 0.8847 | 0 | 5.1267 | 1.0305 | 0.9787 | 3.6235 |
| ***M. majori*** | 4.8114 | 3.947 | 6.3548 | 5.2612 | 1.1999 | 0.6338 | 5.3412 | 5.1267 | 0 | 4.9114 | 5.0351 | 1.5263 |
| ***M. manavi*** | 0.1405 | 1.0071 | 1.4888 | 0.398 | 6.0944 | 5.5181 | 0.4696 | 1.0305 | 4.9114 | 0 | 0.1317 | 3.3884 |
| ***M. petersoni*** | 0.2304 | 1.1382 | 1.3576 | 0.2689 | 6.2196 | 5.6432 | 0.3385 | 0.9787 | 5.0351 | 0.1317 | 0 | 3.511 |
| ***M. sororculus*** | 3.2864 | 2.4387 | 4.8285 | 3.7351 | 2.7203 | 2.1454 | 3.8152 | 3.6235 | 1.5263 | 3.3884 | 3.511 | 0 |

**Table S2**. Euclidean distance matrix of species centroids in morphospace for Malagasy Miniopterus species

**Table S3.** Summary of Euclidean distances among *Miniopterus* species from Kenya and Madagascar. Values represent mean pairwise distances, and smallest and largest observed interspecific distances with standard deviations.

| **Region** | **Mean distance** | **Nearest neighbor** | **Farthest neighbor** |
| --- | --- | --- | --- |
| Continental | 2.6996 ± 0.864 | 0.8075 ± 0.3767 | 5.6635 ± 1.0781 |
| Madagascar | 2.7786 ± 0.965 | 0.5588 ± 0.4669 | 6.3320 ± 0.8164 |
